# Violence against healthcare workers and other serious responses to medical disputes in China: surveys of patients at 12 public hospitals

**DOI:** 10.1186/s12913-020-05104-w

**Published:** 2020-03-26

**Authors:** Yuxian Du, Wenxin Wang, David J. Washburn, Shinduk Lee, Samuel D. Towne, Hao Zhang, Jay E. Maddock

**Affiliations:** 1 Division of Public Health Sciences, Hutchinson Institute for Cancer Outcome Research (HICOR), Fred Hutchinson Cancer Research Center, Seattle, WA 98109 USA; 2 Data Generation and Observational Studies, Bayer Healthcare U.S. LLC, Whippany, NJ 07981 USA; 3 Department of Public Administration, Law School, Shantou University, Shan-Tou, 515063 People’s Republic of China; 4 Department of Health Policy and Management, School of Public Health, Texas A&M University, College Station, TX 77843 USA; 5 Center for Population Health and Aging, Texas A&M University, College Station, TX 77843 USA; 6 Department of Health Management and Informatics, University of Central Florida, Orlando, FL 32816 USA; 7 Disability, Aging, and Technology Cluster Initiative, University of Central Florida, Orlando, FL 32816 USA; 8 Department of Environmental and Occupational Health, School of Public Health, Texas A&M University, College Station, TX 77843 USA; 9 Southwest Rural Health Research Center, Texas A&M University, College Station, TX 77843 USA

**Keywords:** Medical dispute, Violence, Physician-patient relationship, Yi Nao, China, Healthcare reform

## Abstract

**Background:**

Workplace violence against healthcare workers is a global issue that is on the rise, with Chinese healthcare workers facing growing challenges with hospital violence. Attacks on medical staff have increased in recent years with no clear resolution. Prior research focused on policies to improve the doctor-patient relationship and better protect clinicians, but few studies addressed the patient perspective. This paper examines patients’ choices when facing a medical dispute and identifies groups who are more likely to respond to conflict with violence or other serious actions.

**Methods:**

Patient survey responses were collected in 12 leading public hospitals in five Chinese provinces with 5556 participants. The survey asked sociodemographic information, patients’ attitudes (e.g., general optimism, trust in their physicians, perceived healthcare quality), and their primary response to a medical dispute. From least to most severe, the options range from “complaining within the family” to “violence.” We used t-tests and Chi-square tests to explore the relationships between reactions and patient characteristics. We also performed multivariable logistic regressions to determine the impact of sociodemographics and provider trust on the seriousness of responses.

**Results:**

The primary response of a third of respondents was complaining to hospital or health department officials (32.5%). Seeking legal help (26.3%) and direct negotiation with doctors (19.6%) were other frequent responses. More serious responses included 83 stating violence (1.5%), 9.7% expressing a desire to expose the issue to the news media, and 7.4% resorting to seeking third-party assistance. Patients who were more likely to report “violence” were male (OR = 1.81, *p* < .05), high-income earners (OR = 3.71, *p* < .05), or reported lower life satisfaction (OR = 1.40, *p* < .05). Higher trust scores were associated with a lower likelihood of a serious response, including violence (OR = 0.80, *p* < .01).

**Conclusion:**

Most respondents reported mild reactions when facing a medical dispute. Among those who reported the intent of serious reactions, some sociodemographic characteristics and the trust of physicians could be predictive. To prevent future hospital violence, this work helps identify the characteristics of patients who are more likely to seek severe approaches to medical dispute resolution, including resorting to violence. From these results, hospitals will be better able to target specific groups for interventions that build patient-provider trust and improve general patient satisfaction.

## Background

Workplace violence against healthcare workers is a global issue receiving increasing attention [1–4]. This issue is particularly prevalent in China, so much so that it has become a severe public health challenge [5–7]. Between 2000 and 2014, there was an annual increase of 11% in the incidence of violence against medical staff [8]. Between 2000 and 2011, 124 cases of *severe* violence against healthcare workers occurred in Chinese hospitals, including 29 murders and 52 serious injuries [9].

Violence is not limited solely to acts of physical violence but can extend to verbal attacks as well. For example, verbal abuse from patients and relatives has contributed to significant mental stress among healthcare workers [10]. Hospital employees already face high levels of job-related stress and are at-risk for job-related burnout [11]. Thus, the combination of both physical attacks and verbal attacks, either actually carried out or feared, holds serious concern for China’s healthcare workforce as they carry out their already stress-laden and critical role.

The Chinese word, “Yi Nao” (translated as “illegal disturbance and interruption of medical services”), contributes to this public health concern in contemporary China. Yi Nao has evolved so that organized gangs have become involved, seeking out perceived medical malpractice, and violently attacking healthcare providers [12]. Those patients who hire gangs may hope to gain financial compensation from hospitals for the perceived medical malpractice. The anger of the patients and their relatives, along with the threat to healthcare workers, has further exacerbated deteriorating patient-provider relationships and threatens normal functioning within the Chinese healthcare system [13, 14].

Many reasons are cited for this increase in violence against healthcare workers, including long waiting times [9], unrealistic expectations from patients (e.g., complete recovery after one or two visits) [15], limited legal channels for resolving medical disputes [8, 16], and an absence of legal penalties against this violence [8, 17]. Thus, policymakers and others may have no simple solution to this growing and complex issue.

Even though violence might be the most extreme reaction, it is not always the response when medical disputes arise. Opportunities to pursue litigation exist, but research has shown that local courts are insufficiently equipped to handle these matters, leading to plaintiffs having to bring their cases to provincial or central courts to seek resolution, thereby adding extra layers of difficulty [18, 19]. Thus, this may even serve to exacerbate this growing issue further. Given the challenges to a legal resolution, many patients chose to expose their negative experience of medical care to news or media outlets who may actively pursue such headline stories. Previous studies reported that numerous news reports were exaggerated and contributed to deteriorating patient-physician relationships [13]. Other alternatives to medical disputes, including complaints to hospital or healthcare authorities or outside mediation often fail to resolve disputes, because of a lack of adequate mechanisms in place to address patient complaints [17, 20].

While many systematic flaws and causes for medical disputes have been posited, one critical question remains: when patients or patients’ families face a medical dispute, who are the most likely individuals to commit acts of violence against healthcare workers? In response to this gap, the primary aim of this study was to examine the characteristics of those who report being more likely to resort to violence or pursue other serious responses when facing a medical dispute. Those seeking to implement effective interventions that could help address violence in response to disputes should understand the characteristics of those that might be inclined to respond violently. This study would help enable them to carefully design interventions to prevent this behavior while maintaining regular medical services.

## Methods

### Data collection and study participants

Between July and August of 2014, 5714 inpatients and outpatients were recruited from 12 leading public hospitals in metropolitan areas across China. Hospitals were selected in five different provinces (Shandong, Jiangsu, Hubei, Henan, and Sichuan), providing some representation of eastern, central, and western regions of China. In each hospital, inpatients who were able to respond to the survey were included in the survey, while outpatients were asked to participate in waiting rooms. Trained surveyors provided the questionnaires to the participants. Patients were only included in the study if they consented to participate, were literate in Mandarin Chinese, and were coherent when filling out the survey. Almost everyone who was invited agreed to participate, while 20 to 30% did not complete the survey, with some variation among hospitals. Among all survey respondents, those who were born on or before 1999 were selected, thereby reaching a study sample size of 5556.

### Study variables

The dependent variable assessed in this study was the patient’s self-reported response to a medical dispute. We developed the question for this study, asking: “assuming you are facing a medical dispute, what is your primary approach?” The options are listed below, with assigned severity from lowest to highest: (1) complaining to friends and relatives, (2) negotiating with the doctor, (3) complaining to the hospital or the health department, (4) negotiating with a third party_a_, (5) seeking legal help, (6) exposing the issue to the news/media, and (7) resorting to violence. Given the nature of this question, if the patients chose option 7 (resorting to violence), we assume it stands for Type II workplace violence as defined by The National Institute for Occupational Safety and Health (NIOSH) [21, 22], which is violence from customer or client.

We dichotomized the responses into the lower and higher level responses based on their seriousness. The options “complaining to friends and relatives,” “negotiating with the doctor,” and “complaining to the hospital or the health department” were categorized as lower-level responses. The higher-level response category included “negotiating with a third party,” “seeking legal help,” “exposing the issue to the news/media,” and “resorting to violence,” given these actions bring the dispute to an external entity or imply other relatively more severe outcomes. This dichotomization generated relatively balanced samples, with 55 and 45% for lower and higher level responses, respectively.

The independent variables were patients’ opinions on healthcare quality, patient-provider interpersonal trust, and general life attitudes. This survey used the existing composite measures adapted from the Interpersonal Trust Scale (ITS) [23], Wake Forest Physician Trust Scale (WFPTS) [24] and SERVQUAL (Service Quality Scale) [25], to evaluate the quality of care [26] and patient-physician trust [27, 28]. We also developed specific questions on participants’ attitudes towards their life and health. General attitudes measures and standard scales measuring trust and quality all ranged from 1 to 5, with 1 as the worst (e.g., lowest trust, lowest satisfaction, etc.), and 5 as the best (e.g., highest perceived quality, most optimistic). Each survey also included standard questions on sex, year of birth, marital status, education level, occupation, rural or urban residence, insurance type, and the individuals’ income level. Considering the possible impact from traveling distance and transportation difficulty, we designed a question in the survey asking whether patients were from the same county as the hospital they were seeking care in. The possible answers included the same county, out of the county, but within the province, and out of the province. Other general healthcare utilization questions included the type of services for which they sought care (inpatient or outpatient), previous experience with the hospital (having been to the very hospital or not), as well as their perception about healthcare quality received (1-worst, 5-best, Likert scale) and their trust towards this hospital and their physicians (1-worst, 5 best, Likert scale). These questions sought to investigate whether there were associations between healthcare utilization and their attitude when facing disputes.

### Data analyses

Chi-squared tests and t-tests were used to describe bivariate relationships between the independent variables and responses to approaches when dealing with medical disputes in two categories (lower or higher severity). The responses for all independent variables were broken down by types of reactions to medical disputes, comparing lower-level to higher-level, as well as non-violent to violent responses.

We used two sets of multivariable logistic regression analyses to assess the potentially influential factors in patient responses to medical disputes. One used the binary outcome variable of higher vs. lower severity, while the other used violent vs. all other non-violent responses. Independent variables included demographic characteristics, socioeconomic status, and attitudes towards trust and service quality as separate sets of independent variables for the two regression models. Key demographic variables (age, sex, a first-time visitor or not, and inpatient or outpatient) were included in both models as controls regardless of their significance in bivariate analysis. Other variables that had *p* < .10 in the bivariate analyses were included in the regression analyses using a forward selection method. The significance level of 0.05 was used for the regression analyses. All analyses were conducted using SAS 9.4 (Cary, NC).

## Results

### Categories of responses to medical disputes

Table 1 describes the frequency distribution for participants’ primary response to a medical dispute. Among those with lower-level (less severe) responses, 165 individuals (overall 3%) chose “complaining to friends and relatives,” which may mean not taking any actions to resolve the medical dispute. In addition, about 20% of the respondents chose “negotiating with the doctor,” and nearly 33% of the participants selected “complaining to the hospital or the health department,” which was the most common response. For those with higher-level responses, over 26% of all participants picked “seeking legal help,” 7% of the respondents would negotiate with a third party. A significant group of respondents also gave more serious responses, including “exposing to the news/media” (9.7%) and “resorting to violence” (1.5%).

**Table 1:** Frequencies of answers to the potential approaches for medical dispute

Options when facing a medical dispute	Frequency	Percentage	Categorization
Complaining to friends and relatives	165	3.0%	Lower-level Response (*N* = 3059; 55.1%)
Negotiating with the doctor	1087	19.6%	
Complaining to the hospital or the health department	1807	32.5%	
Negotiating with a third party	413	7.4%	Higher-level Response (*N* = 2497; 44.9%)
Seeking legal help	1461	26.3%	
Exposing to the news/media	540	9.7%	
Resorting to violence	83	1.5%	
**Total**	**5556**	**100.0%**	

### Bivariate analysis: sociodemographic characteristics, attitudes, and trust and quality scales

Three thousand fifty-nine participants (55.1%) reported lower-level (less severe) responses, and 2497 participants (44.9%) reported higher-level (more severe) responses. Their sociodemographic information is shown in Table 2, along with bivariate analyses by (1) response severity level (lower vs. higher) and (2) violence vs. non-violence. The different categories of responses to medical disputes had variation in the distribution of sociodemographic characteristics.

**Table 2:** Sociodemographic characteristics of participants by response to medical dispute

Frequency (Column %)	Lower-level Response (***n*** = 3059)	Higher-level Response (***n*** = 2497)	p	Non-violence (***n*** = 5473)	Violence (***n*** = 83)	p
**Sex**						**
Female	1667 (54.5%)	1342 (53.7%)		2978 (54.4%)	31 (37.3%)	
Male	1392 (45.5%)	1155 (46.3%)		2495 (45.6%)	52 (62.7%)	
**Birth Year**						†
1949 or before	317 (10.4%)	258 (10.3%)		568 (10.4%)	7 (8.4%)	
1950–1959	292 (9.5%)	230 (9.2%)		518 (9.5%)	4 (4.8%)	
1960–1969	437 (14.3%)	322 (12.9%)		751 (13.7%)	8 (9.6%)	
1970–1979	570 (18.6%)	541 (21.7%)		1094 (20.0%)	17 (20.5%)	
1980–1989	957 (31.3%)	809 (32.4%)		1732 (31.6%)	34 (41.0%)	
1990–1999	486 (15.9%)	337 (13.5%)		810 (14.8%)	13 (15.7%)	
**Marital Status**			**			
Unmarried	633 (20.7%)	448 (17.9%)		1061 (19.4%)	20 (24.1%)	
Married	2426 (79.3%)	2049 (82.1%)		4412 (80.6%)	63 (75.9%)	
**Education**			*			
Junior High School or Below	1119 (36.6%)	981 (39.3%)		2072 (37.9%)	28 (33.7%)	
Senior High School or equivalent	871 (28.5%)	704 (28.2%)		1550 (28.3%)	25 (30.1%)	
Junior College or Higher	1069 (34.9%)	812 (32.5%)		1851 (33.8%)	30 (36.1%)	
**Unemployed**	806 (26.3%)	728 (29.2%)	†	1514 (27.7%)	20 (24.1%)	
**Travel distance measure**			*			
Short-distance	2439 (79.7%)	2048 (82.0%)		4419 (80.7%)	68 (81.9%)	
Mid-distance	406 (13.3%)	300 (12.0%)		695 (12.7%)	11 (13.3%)	
Long-distance	214 (7.0%)	149 (6.0%)		359 (6.6%)	4 (4.8%)	
**Rural**	1665 (54.4%)	1449 (58.0%)	**	3067 (56.0%)	47 (56.6%)	
**Urban**	1394 (45.6%)	1048 (42.0%)		2406 (44.0%)	36 (43.4%)	
**Monthly Income (CNY)**						†
< 1000	636 (20.8%)	552 (22.1%)		1170 (21.4%)	18 (21.7%)	
1000 ~ 1999	676 (22.1%)	560 (22.4%)		1220 (22.3%)	16 (19.3%)	
2000 ~ 2999	733 (24.0%)	617 (24.7%)		1336 (24.4%)	14 (16.9%)	
3000 ~ 4999	709 (23.2%)	523 (20.9%)		1212 (22.1%)	20 (24.1%)	
5000 ~ 9999	257 (8.4%)	193 (7.7%)		441 (8.1%)	9 (10.8%)	
> = 10,000	48 (1.6%)	52 (2.1%)		94 (1.7%)	6 (7.2%)	
**Inpatient**	1687 (55.1%)	1392 (55.7%)		3044 (55.6%)	35 (42.2%)	*
**Outpatient**	1369 (44.8%)	1105 (44.3%)		2426 (44.3%)	48 (57.7%)	

Mantel-Haenszel Chi-Square Test *p*-value indicator: “†” *p* < .10, * *p* < .05, ** *p* < .01, *** *p* < .001. This test examines the alternative hypothesis that there is a linear association between the two variables. The item “travel distance measure” was derived from a question asking patients’ hometown locations relative to the hospital location. Three options were provided in the multiple-choice question: within the same county, outside of the county within the same province, and outside of the province. These three options were designated “short-distance,” “mid-distance,” and “long-distance,” respectively

Significant differences between a lower-level response and a higher-level response to medical disputes were found for marital status, education, travel distance, and rural-urban status. Compared to those who reported lower-level responses, participants reporting a higher-level response were more likely to be married (versus not married, *p* < .01), educated at the junior high school level or below (versus senior high school or equivalent or higher, *p* < .05), unemployed (versus employed, *p* < .10), traveling within the county (versus outside the county or province, *p* < .05) and residing in a rural area (versus an urban area, *p* < .01).

For the comparison between those who chose non-violent options (*n* = 5473) versus violent responses (*n* = 83), statistically significant variations were found for characteristics including sex, age, income, and outpatient status. While the group reporting that they would pursue violent responses to medical disputes was small, those who disproportionally reported violence as a preferred option to disputes were more likely to be male (*p* < .01), younger (*p* < .10), reporting relatively higher income (*p* < .10), and outpatients (*p* < .05). Those who were born in the 1980s (*p* < .10) and 1990s (*p* < .10) reported a higher likelihood of pursuing violence as their primary response to a medical dispute. Individuals within the highest tier of income (> = CNY 10,000 per month) had a 7.2% share of the “resorting to violence” responses, but only 1.7% share of the non-violent response (*p* < .10).

As shown in Table 3, the trust and quality scales showed statistically significant bivariate association with participants’ primary response to a medical dispute, both in lower vs. higher level comparisons and in violence vs. non-violence response comparisons (all *p* < .01).

**Table 3:** General attitudes and standard scale measures by response to medical dispute

Mean (Standard Deviation)	Lower-level Response (***n*** = 3059)	Higher-level Response (***n*** = 2497)	p	Non-violence (***n*** = 5473)	Violence (***n*** = 83)	p
**Attitudes**						
Satisfaction with Life	3.52 (0.77)	3.44 (0.79)	***	3.49 (0.78)	3.16 (0.90)	**
Perceived Importance of Health	4.10 (0.74)	4.10 (0.74)		4.10 (0.74)	3.92 (0.83)	*
General Optimism about Life	3.80 (0.72)	3.78 (0.74)		3.79 (0.73)	3.65 (0.82)	†
**Trust and Quality Scales**						
WFPTS	3.39 (0.65)	3.29 (0.68)	***	3.35 (0.67)	2.96 (0.67)	***
ITS	2.69 (0.36)	2.65 (0.37)	***	2.68 (0.36)	2.53 (0.44)	**
SERVQUAL	3.70 (0.57)	3.64 (0.57)	***	3.68 (0.57)	3.36 (0.65)	***

“†” *p* < .10, * *p* < .05, ** *p* < .01, *** *p* < .001. All items in this table were measured on a scale from 1 to 5, with 1 as the worst state of being, and 5 as the best state of being. The *p*-values in Table 3came from the two-sample t-test. [Abbreviations] *WFPTS* Wake Forest Physician Trust Scale, *ITS* Interpersonal Trust Scale, *SERVQUAL* Service Quality Scale

Those individuals who selected a higher-level response to medical disputes had lower trust scores from both physician trust (WFPTS) and interpersonal trust (ITS), as well as lower perceptions of service quality (SERVQUAL). Similar trends also appeared in the group selecting violence as a response to disputes, with lower scores (*p* < .001): WFPTS at 2.96, ITS at 2.53, and SERVQUAL at 3.36, comparing to 3.35, 2.68 and 3.68 in the non-violent group, respectively. Participants who reported higher-level responses were less-satisfied with life than those who reported lower-level responses (*p* < 0.001). In addition, “satisfaction with life” (*p* < .01), “perceived importance of health” (*p* < .05), and “general optimism about life” scores (*p* < .10) were significantly different among those would resort to violence versus others.

### Multivariate Logistic Regressions

For comparisons between higher- and lower-levels of responses to a medical dispute, influential factors were travel distance, rural-urban status, satisfaction with life, and both trust scales in the multivariate analysis (Table 4). As relative travel distance increased (i.e., traveling outside of the county or province), patients’ tendency to report a higher-level response decreased, but no statistical significance was found. Rural residents were more likely to select the higher-level responses when compared to urban residents (OR = 1.167, *p* < .05). As trust scores (both WFPTS and ITS) increased, the likelihood of reporting higher-level responses to a medical dispute decreased (WFPTS: OR = 0.808, *p* < .001; ITS: OR = 0.796, *p* < .01).

**Table 4:** Logit regression model on reactions towards medical dispute (95% CI)

	Model 1: Higher vs. Lower Level Response		Model 2: Violence vs. Other Response	
***Sociodemographics***	**OR**	**95% CI**	**OR**	**95% CI**
**Female (ref: Male)**	0.988	(0.881, 1.109)	0.551*	**(0.339, 0.894)**
**Birth Year**	(Ref: 1949 or before)			
1950–1959	0.920	(0.719, 1.176)	0.571	(0.164, 1.984)
1960–1969	0.891	(0.709, 1.120)	0.839	(0.295, 2.390)
1970–1979	1.146	(0.921, 1.425)	1.139	(0.448, 2.897)
1980–1989	1.027	(0.827, 1.277)	1.242	(0.509, 3.028)
1990–1999	0.977	(0.744, 1.283)	0.958	(0.343, 2.678)
**Marital Status**	(Ref: unmarried)			
Married	1.089	(0.915, 1.296)	0.890	(0.473, 1.678)
**Travel Distance Measure**	(Ref: Short-distance)			
Mid-distance	**0.852†**	**(0.719, 1.010)**	*(Not included)*	
Long-distance	0.833	(0.662, 1.047)		
**Rural (ref: Urban)**	1.167*	**(1.027, 1.326)**	*(Not included)*	
**Education**	(Ref: <= Junior High)			
Senior High School or equivalent	0.934	(0.806, 1.084)	*(Not included)*	
Junior College or Higher	0.910	(0.771, 1.074)		
**Unemployed (ref: employed)**	1.056	(0.923, 1.209)	*(Not included)*	
**Monthly Income (CNY)**	(Ref: 2,000 ~ 2999)			
< 1000	*(Not included)*		**1.905†**	**(0.904, 4.017)**
1000 ~ 1999			1.504	(0.722, 3.134)
3000 ~ 4999			1.341	(0.667, 2.697)
5000 ~ 9999			1.540	(0.648, 3.658)
> = 10,000			3.712*	**(1.328, 10.381)**
***Experience***				
First-time visitor (ref: non-1st-timer)	1.004	(0.876, 1.150)	0.868	(0.505, 1.490)
Inpatient (ref: Outpatient)	1.047	(0.926, 1.185)	0.780	(0.926, 1.185)
***Attitudes***				
Satisfaction with Life	**1.066†**	**(0.990, 1.148)**	1.404*	**(1.031, 1.911)**
Perceived Importance of Health	*(Not included)*		1.206	(0.901, 1.616)
Optimism Measure	*(Not included)*		0.908	(0.648, 1.273)
***Trust and Quality Scales***				
WFPTS	0.808***	**(0.720, 0.905)**	0.719	(0.460, 1.123)
ITS	0.796**	**(0.678, 0.936)**	0.638	(0.351, 1.159)
SERVQUAL	1.002	(0.881, 1.139)	0.675	(0.421, 1.084)

“†” *p* < .10, * *p* < .05, ** *p* < .01, *** *p* < .001. Certain variables were included in the multivariate regression regardless of their statistical significance in bivariate analysis. These variables are age, gender, marital status, a first-time visitor or not, inpatient or outpatient. Other covariates were “not included” if they were not statistically significant at 90% confidence level (*p* > = .10, as indicated in the table). The odds ratios (O.R.s) here are adjusted odds ratios (AORs), meaning that they are controlled for other predictors in the model

For comparisons between violent and non-violent responses to medical disputes, influential factors included sex and income (Table 4). Patients who reported higher levels of life satisfaction were also more likely to report “resorting to violence” when facing a medical dispute (OR = 1.404, *p* < .05). Females were less likely to select violence as an option than males (OR = 0.551, *p* < .05). Compared to a monthly income of CNY 2000 ~ 2999, the highest tier incomes (> = CNY 10,000) were more likely to select violence (OR = 3.712, *p* < .05).

## Discussion

This study adds a critical assessment, to the insufficiently studied existing literature, of factors associated with hospital violence and other extreme responses related to medical disputes. Further, we provide a unique patient perspective from a low-to-middle income country experiencing major policy-related changes within the health care system in recent years, namely health care financing reform [26, 29, 30]. Apart from the influence of trust of physicians and perceived healthcare quality in bivariate analysis, we were able to find associations between sociodemographic characteristics and more extreme responses related to medical disputes, including violence.

This study highlights several underlying factors related to more severe responses to medical disputes. Those who resided in rural areas tended to choose a more severe type of response when faced with a medical dispute. Some rural respondents may have limited knowledge or experience related to non-violent or other less severe alternatives when facing a medical dispute or otherwise negative experience. Those with the highest income had a higher likelihood of “resorting to violence.” Providing informal payments to physicians in the forms of red envelop or gifts is not uncommon in Chinese culture [31]. Further, some research argues that the association between health inequity and poverty may affect the healthcare experiences between low-income and high-income patients [32]. Future research is needed to study the impact of income and provide a more theoretical framework explaining the violence tendency in different income groups.

In this study, we also found that participants who reported greater satisfaction with their life were more likely to report violence as their primary response to a medical dispute. This paradoxical result suffers from selection bias and should be interpreted with caution. Considering the skewness of the responses to the “life satisfaction” question (more than 80% answering “satisfied” or “ordinary”) and only 1.5% (*n* = 83) reporting ‘resorting with violence, it may not be unusual to observe more individuals reporting “violence” in the “satisfied” or “ordinary” categories than that in the “dissatisfied” or “very dissatisfied” categories.

While the supply side shortage remains a possible cause of violence against healthcare workers, patients’ expectations could be another critical factor. An outcome from a tendency in patients to seek care, early on, at the hospital level is that patients have unrealistic expectations of the efficiency of secondary and tertiary care providers. Examples of these unrealistic expectations include receiving all the medications they want even if not medically indicated, obtaining an immediate and complete recovery, or a considerable improvement, all from a single, long-awaited visit [13]. When the prescription, diagnosis, or outcome is different from their expectations, patients and their relatives could perceive that as medical malpractice and quickly become anxious or even angry.

The current study also found that patients’ trust in their physicians were strongly associated with their selected primary reaction in a medical dispute. Challenges within the Chinese healthcare system seem to be related to the underlying factor associated with patients’ responses to engage in violence when facing a medical dispute. Since the commercialization of medicine in 1985, public hospitals in China have not had consistent government funding, so they may have sought other approaches (e.g., over-prescribing medication and other treatments) to offset the costs [6]. While patients bear the burden of paying higher prices for medical services that are exceeding the current inflation rate, healthcare workers, especially physicians, are getting salaries far less than their counterparts in other countries. While patients may have a negative experience within the healthcare system, providers are also facing double challenges of low pay and high workload [33]. Further, biased media reports and distorted social norms are negatively affecting healthcare workers, as news reports typically focus on negative events in which patients are characterized as victims from medical malpractice [8, 34]. These factors likely contribute to deteriorating relationships between patients and healthcare workers.

Studying violence against healthcare workers is critical because this violence could have many detrimental effects, which could further harm the patient-provider relationship and generate a higher likelihood of future violence or extreme behaviors. Violence against medical personnel can increase the psychological stress of healthcare workers [35, 36], and drive individuals away from the medical profession [33], further reducing the healthcare workforce and making it harder to meet patients’ demands. With one-fifth of the world’s population (1.4 billion) residing in China [37], the available workforce in the healthcare sector is far lower when compared to other countries. In 2014, China had 1724 physicians per 1 million population, while the United States had 2568, and Cuba had 7519 as the world’s highest [38]. Furthermore, patients in China can seek secondary and tertiary care providers without a referral from primary care providers. Thus, large public hospitals that house many of the secondary and tertiary providers often face crowds far beyond their limited capacity, leading to extremely long waiting times [39]. This no-referral-needed system further tilts the balance between supply and demand in the healthcare delivery system and worsens the issue of workforce shortages in China. The possible link between workplace violence, high demand, and understaffing in China could be impactful, as this association has already been demonstrated in some European studies [3, 22]. Further-more, given legal channels for resolving confusion or anxiety are sometimes lacking and there is difficulty in implementing penalties for violence [17], verbally or physically attacking the healthcare worker becomes a way to release patients’ anger [12, 16]. That said, policies aimed at promoting the role of general practitioners in primary care have been suggested as a way to bring about positive change to the existing system through improved access to care [40].

Financial challenges within the healthcare system, such as physicians not receiving sufficient salary and patients paying too much for medical care, could also be contributing to the worsening patient-physician relationship and may ultimately lead to patients’ extreme actions [6, 33]. Further, current interventions are both limited in number and effectiveness. Police departments have been sending more armed security guards to at-risk hospitals, while multiple studies argue that this does not improve weak provider-patient trust and, in fact, maybe more detrimental than doing nothing at all [7, 8]. Regulatory agencies are mandating price controls over medical services, which could make it difficult for hospitals to meet rising costs. As the economic pressure accumulates on both the providers’ and the patients’ sides, the gap between patient expectation and healthcare services quality and cost may be diverging further. Therefore, reforms at the national level, targeted at reducing economic burdens on the entire healthcare delivery system, are essential to improving the patient-provider relationship and reducing the incidence of violence and other extreme responses when medical disputes arise. Other reforms may also hold promise. For example, the National Hierarchical Medical System, among other things, includes policies relating to promoting the role of general practitioners. However, past research has noted a relatively limited awareness of this policy and the role of general practitioners [40]. Thus, providing greater awareness of the role of general practitioners and the National Hierarchical Medical System has been suggested [40] as a means of addressing health care access issues, to an extent, given the relatively underutilized primary care settings throughout China.

## Limitations

This study has limitations in generalizability and from self-report. Even though the survey received more than 5000 responses, the findings cannot be generalized to all patients in China, given its geographic and demographic diversity. Moreover, this survey only included respondents in large public hospitals because of their large capacity and diversity in patients seeking care at these facilities, rather than primary care facilities or private hospitals. The generalizability of this study is also limited in that the survey question did not specify which healthcare providers (e.g., physicians, nurses, administrative staff, etc.) were the potential recipients of violence. While the lack of specific detail about which healthcare providers were potential targets may encourage some respondents to provide a relatively honest answer, further studies by types of healthcare providers are necessary.

Another limitation of this study is reporting bias. First, participants recruited in this study were able to read and speak Mandarin, which was necessary given the survey language (Mandarin). This may have led to the recruitment of those with at least minimal literacy needed to complete the survey and speak with study staff. As such, those who did not read or speak Mandarin or those who were illiterate may have been underrepresented holding potential implications as one’s level of literacy may be associated with aggressive tendencies [41, 42]. Second, realistically, patients may be unlikely to report their tendency towards violence when responding to a survey, especially when the survey was conducted in the very hospital where they seek care. Therefore, this study might have underestimated the number of patients whose primary reaction to a medical dispute is violence. The low number of patients responding “resorting to violence” (*n* = 83 out of 5556) in this study also limits our power to detect possible factors associated with a tendency towards violence.

In addition, given practical limitations in terms of available resources (e.g., funding, time), we were unable to carry out a mixed-method approach (e.g., purposively sampling from those reporting a tendency towards violence) that would have complemented the survey findings with more in-depth interviews. Future research should consider the benefits and limitations of using qualitative interviews for potentially sensitive questions and prepare appropriate methods to address potential bias in reporting.

## Conclusions

This is one of the first studies investigating the impact of patient characteristics on their response to medical disputes, especially regarding the issue of violence against healthcare workers in China. We suggested a model in examining the possibility of resorting to higher-level reactions to medical disputes, which include socioeconomic status, perceived healthcare quality, and potential risks of extreme behaviors. Although further investigation is necessary to provide reliable guidance, those seeking to implement effective and targeted interventions should focus on these indicators for possible extreme behaviors related to medical disputes. Hospitals should continue to improve their quality of care and trust between patients and healthcare workers. Populations with relatively lower socioeconomic status may need additional assistance so that they may meet their basic needs for healthcare, and patients feel greater satisfaction with their health care providers and the health care services they receive.

As shown in the existing literature, critical stakeholders at organizational and state levels, including but not limited to legal administration and enforcement agencies, must be involved in preventing and addressing violence towards health care workers [43]. The legal system should develop appropriate penalties for individuals who have violent behaviors towards healthcare workers. The active participation of all aspects of society could reduce the incidence of extreme reactions towards medical disputes and prevent them from developing into Yi Nao. Aggression and violence against healthcare workers are prevalent in other countries without Yi Nao as well [4, 44], and as such, stakeholders in China may be able to learn from other examples of how to address this issue with a systematic reform in the healthcare framework [35, 36]. If the tense relationship between healthcare providers and patients can be improved, psychological pressures on healthcare workers may be alleviated, and China will have a much more sustainable healthcare workforce, which is critical to sustainably improve healthcare service delivery.

## Data Availability

The datasets generated and/or analyzed during the current study are not publicly available due to data protection programs administered by the National Science Foundation of China but are available from the corresponding author on reasonable request.

## Abbreviations

CDC: Centers for Disease Control and Prevention
ITS: Interpersonal Trust Scale
NIOSH: The National Institute for Occupational Safety and Health
OR: Odds Ratio
WFPTS: Wake Forest Physician Trust Scale
SERVQUAL: Service Quality Scale
